# Different spatial patterns of nitrogen and phosphorus resorption efficiencies in China’s forests

**DOI:** 10.1038/s41598-017-11163-7

**Published:** 2017-09-06

**Authors:** Shan Xu, Guoyi Zhou, Xuli Tang, Wantong Wang, Genxu Wang, Keping Ma, Shijie Han, Sheng Du, Shenggong Li, Junhua Yan, Youxin Ma

**Affiliations:** 1 South China Botanical Garden, Chinese Academy of Sciences, Guangzhou, 510650 China; 2 College of Tourism, Henan Normal University, Xinxiang, 453007 China; 3 Institute of Mountain Hazards and Environment, Chinese Academy of Sciences, Chengdu, 610041 China; 4 State Key Laboratory of Vegetation and Environmental Change, Institute of Botany, Chinese Academy of Sciences, Beijing, 100093 China; 5 Key Laboratory of Forest Ecology and Management, Institute of Applied Ecology, Chinese Academy of Sciences, Shenyang, 110016 China; 6 State Key Laboratory of Soil Erosion and Dryland Farming on Loess Plateau, Institute of Soil and Water Conservation, Chinese Academy of Sciences and Ministry of Water Resources, Northwest Agriculture and Forestry University, Yangling, 712100 China; 7 Key Laboratory of Ecosystem Network Observation and Modelling, Institute of Geographic Sciences and Natural Resources Research, Chinese Academy of Sciences, Beijing, 100101 China; 8 Xishuangbanna Tropical Botanical Garden, Chinese Academy of Sciences, Mengla, 666303 China

## Abstract

Nutrient resorption is an important internal-strategy for plant to retain nutrients. However, the spatial patterns of nitrogen and phosphorus resorption efficiencies (NRE, PRE) in national scales are still unexplored. In this study, we first estimated the magnitudes of NRE and PRE, and explored their spatial patterns across China’s forests based on the dataset from a nation-wide field campaign from 2011 to 2015. Mean NRE was estimated to be 35.64% and higher than mean PRE (43.72%). The main effects of forest type and the interactions between climatic zone and land use were significant for both NRE and PRE. In addition, NRE and PRE exhibited different patterns along climatic gradients and nutrient status. Our results can shed light on the nutrient strategies of China’s forests under future environmental changes and the results could be used in global biogeochemical models.

## Introduction

Nutrient availability plays a key role in forest carbon (C) dynamics and the efficiency of biomass accumulation^1, 2^, especially the availability of macronutrients, e.g. nitrogen (N) and phosphorus (P)^3^. Apart from the uptake of nutrients by roots^4^ and their associated mycorrhizal fungi^5^, plants also transfer soluble nutrients from old leaves to other tissues through the phloem before abscission, which is defined as nutrient resorption^6, 7^. Nutrient resorption is an important internal-strategy for plant to retain nutrients^8^, and has been estimated to account for 31% and 40% of plant N and P demands on average at the global scale^9^. Both nitrogen resorption efficiency (NRE) and phosphorus resorption efficiency (PRE) have been assumed to be 50% in most models, whereas in a global meta-analysis, NRE and PRE were estimated to be 62.1% and 64.9%, respectively^10^. Given the important role of resorption in plant nutrient supply, exploration of their spatial patterns and the drivers is urgent and important for predicting forest productivity.

Leaf habit, developmental stage, climate and soil fertility could influence the magnitude of NRE and PRE^7^. Although the factors regulating the magnitude of NRE and PRE in woody plants are similar, the responses of NRE and PRE to various factors were found to be distinct^8, 11^. For example, NRE has been found to be higher in deciduous species than in evergreen species^12^. It increased with latitude, but was negatively related with mean annual temperature (MAT) and mean annual precipitation (MAP) at a global scale^8^. In addition, the magnitude of NRE was closely related with plant functional types, but climate and soil types played minor roles^7, 12^. It was also found to be not affected by soil P content^11^. However, PRE has been found to be generally higher in evergreen species than in deciduous species^12^, and decreased with latitude but increased with MAT and MAP^8^. It was heavily impacted by the content of soil N in a temperate forest, and closely related with climatic and edaphic variable^7, 11, 12^. The inconsistencies of NRE and PRE patterns in previous studies could be explained by the differences in the forms of N and P both above- and belowground, in which a larger proportion of N than P was bound in structural compounds. For aboveground, a large proportion of N in plants are in macromolecules in chloroplasts or occluded in cell wall^13^; Whereas, a greater fraction of P in plants is in inorganic forms, which determines that the process of P resorption is less energy-taking in comparison with N^14^. Differences in chemical forms of N and P can lead to different susceptibilities of N and P to foliar leaching^13, 15^, and affect the preservation of nutrients in leaves. Secondly, for belowground, N and P have different ways of being used by plants. The pathways of N fixation^16^, deposition^17^, mineralization^18^ and removal processes, such as anammox and denitrification^19^, can contribute to soil N availability. However, the availability of P heavily relies on rock weathering, and the input rate is relatively low^3^. Phosphorus in soil is much less mobile and soluble compared to N^20, 21^. The differences in the status of N and P in terrestrial ecosystems probably lead to different patterns and mechanisms of NRE and PRE, and will affect nutrient concentrations in litter and subsequently affect the nutrient released from litter. The patterns and mechanisms of NRE and PRE in forests along natural gradients of nutrient availability, particularly at a national scale are still unclear and further investigation is needed^7^.

China’s forests encompass broad climatic regions, including (sub-)tropical and temperate zones, under contrasting environments^22^. Over the last three decades, deforestation and reforestation have led to China’s forests into different stand stages^22^. The forests in China are undergoing severe N deposition and its average rate has been increasing from 13.2 kg/ha to 21.1 kg/ha during last two decades^23^, and has largely affected soil N and P cycling^24^. Factors related with the diversity of China’s forests, including climatic zones^8, 10^, leaf traits^25^, nutrient status^26, 27^ and stand stages^28^, probably affect NRE and PRE, and provide an ideal opportunity to natural gradients to investigate the spatial patterns of NRE and PRE in a national scale.

We conducted a nation-wide field campaign from 2011 to 2015 to collect mature leaf, litter and soil in 0–10 cm to estimate the magnitude of NRE and PRE and explore their spatial patterns in forests of China. We tried to address the following questions: (1) Whether NRE and PRE share a similar spatial pattern at the national scale? (2) What are the differences between NRE and PRE under different climatic zones, forest types and land uses? (3) How do environmental gradients affect and regulate NRE and PRE in forest ecosystem?

## Results

### Variations in NRE and PRE in climatic zone, forest type and land use

**Figure 1:**
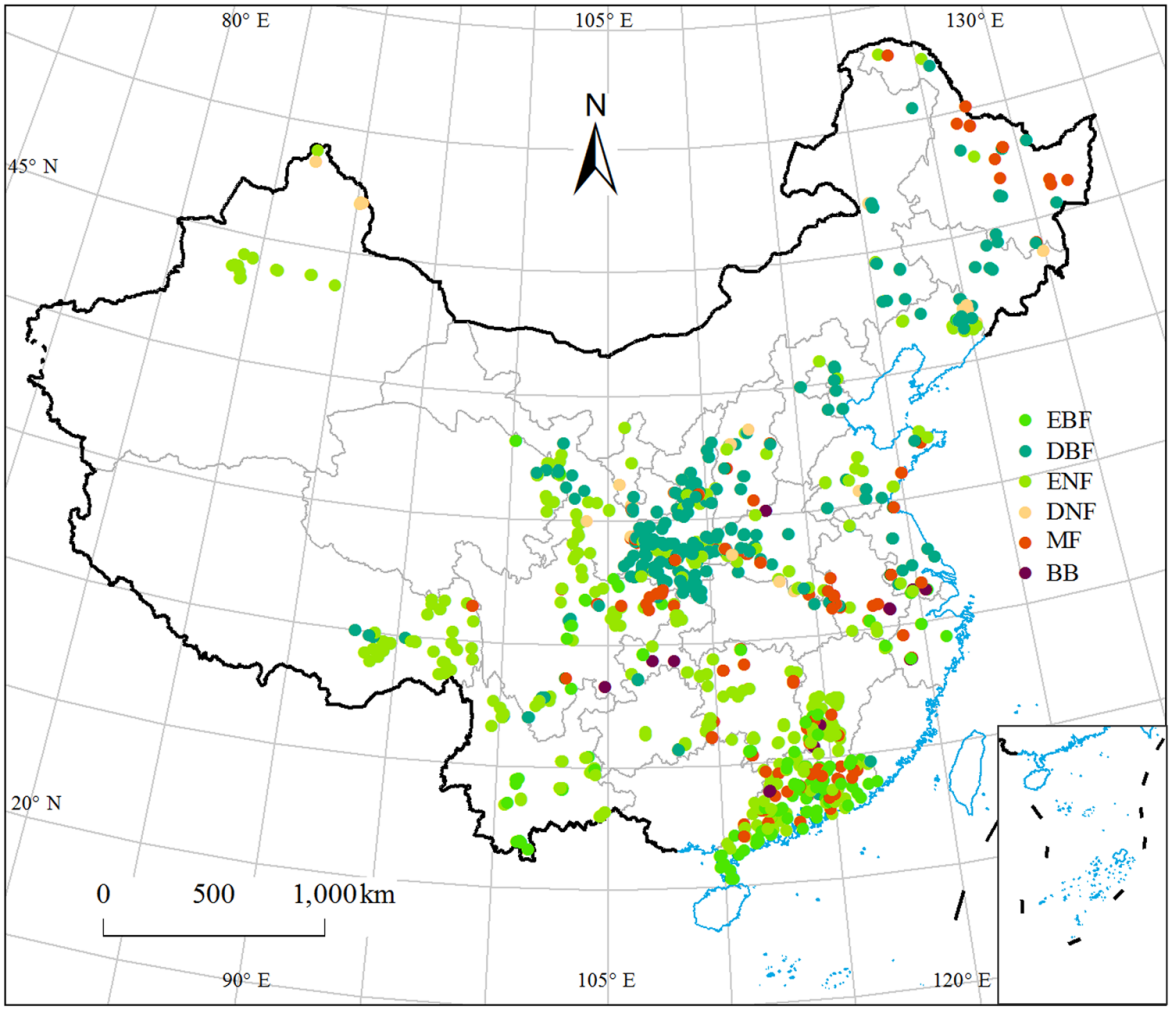
The distributions of sampling sites in China’s forests (Map created using ARCGIS 10.1 software by fourth author, URL: http://www.esri.com). EBF: evergreen broadleaf forest, DBF: deciduous broadleaf forest, ENF: evergreen needle-leaf forest, DNF: deciduous needle-leaf forest, MF: broadleaf and needle-leaf mixed forest, BB: bamboo forest.

**Figure 2:**
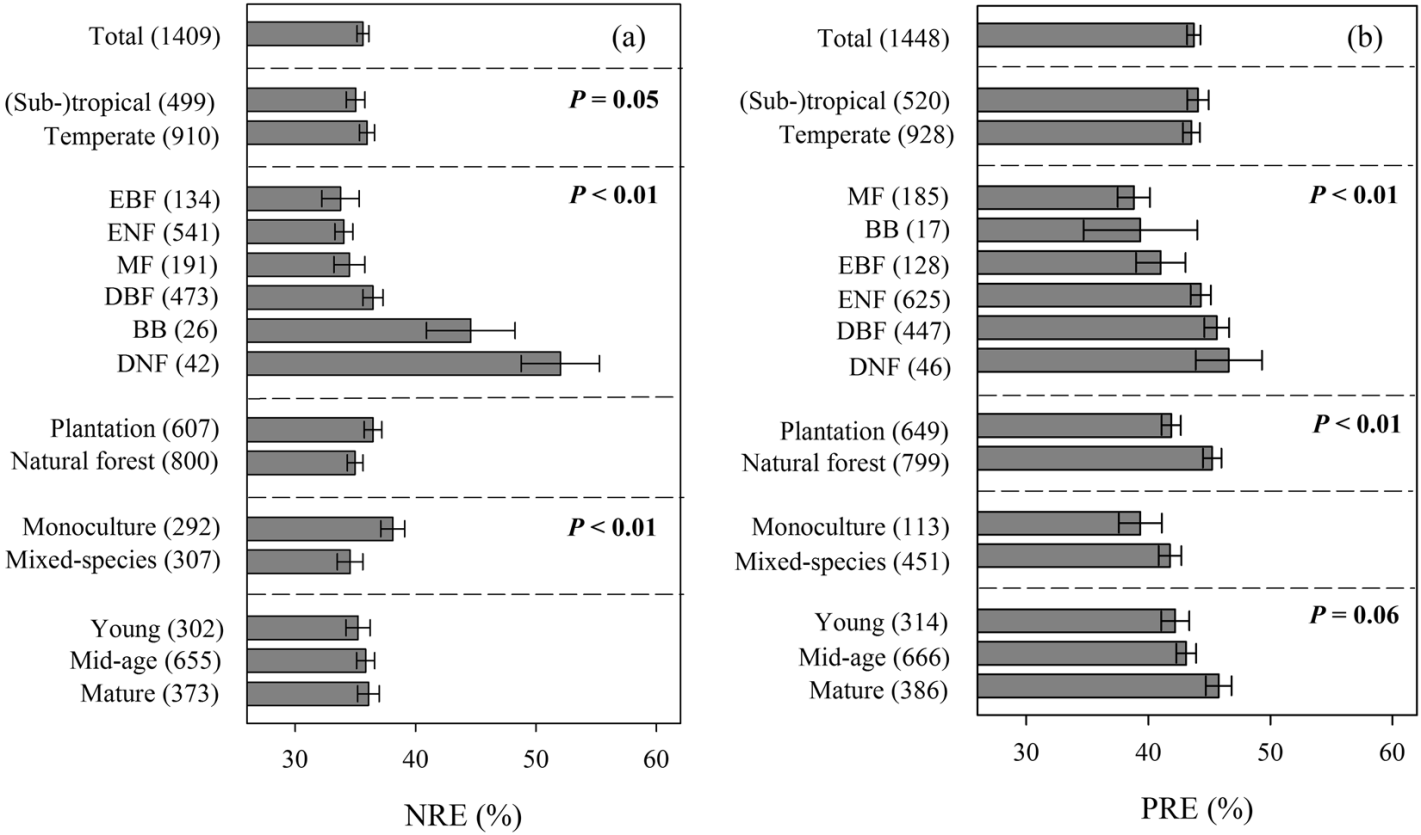
Mean NRE and PRE according to different classified standards. (**a**) Mean NRE across China’s forests, different climatic zones, different forest types, plantation and natural forests; (**b**) Mean PRE across China’s forests, different forest types, plantation and natural forests, and different stand stages. NRE: N resorption efficiency, PRE: P resorption efficiency, Young: forest in young stage, Mid-age: forest in mid-age stage, Mature: forest in mature stage. Abbreviations of forest types are the same as Fig. 1. The error bars represent the standard error. The main effects were significant when *P* < 0.05.

Mean NRE and PRE were 35.64 ± 0.48% (n = 1409) and 43.72 ± 0.55% (n = 1448), respectively (Fig. 1 and 2a,b). The resorption efficiency of N varied dramatically among climatic zones and forest types (Fig. 2a and Table S1). It increased as in the following forest types: evergreen broad-leaved forest (EBF) < evergreen needle-leaved forest (ENF) < deciduous broad-leaved forest (DBF) < deciduous needle-leaved forest (DNF) (Fig. 2a). Significant differences of PRE were observed among forest types and types of land use, and marginally significant difference among stand stages (Fig. 2b and Table S1). It appeared in the following order among forest types: EBF < ENF < DBF < DNF. The resorption efficiency of P in plantation is lower than in natural forest, and PRE was marginally highest in mature forests (Fig. 2b).

**Figure 3:**
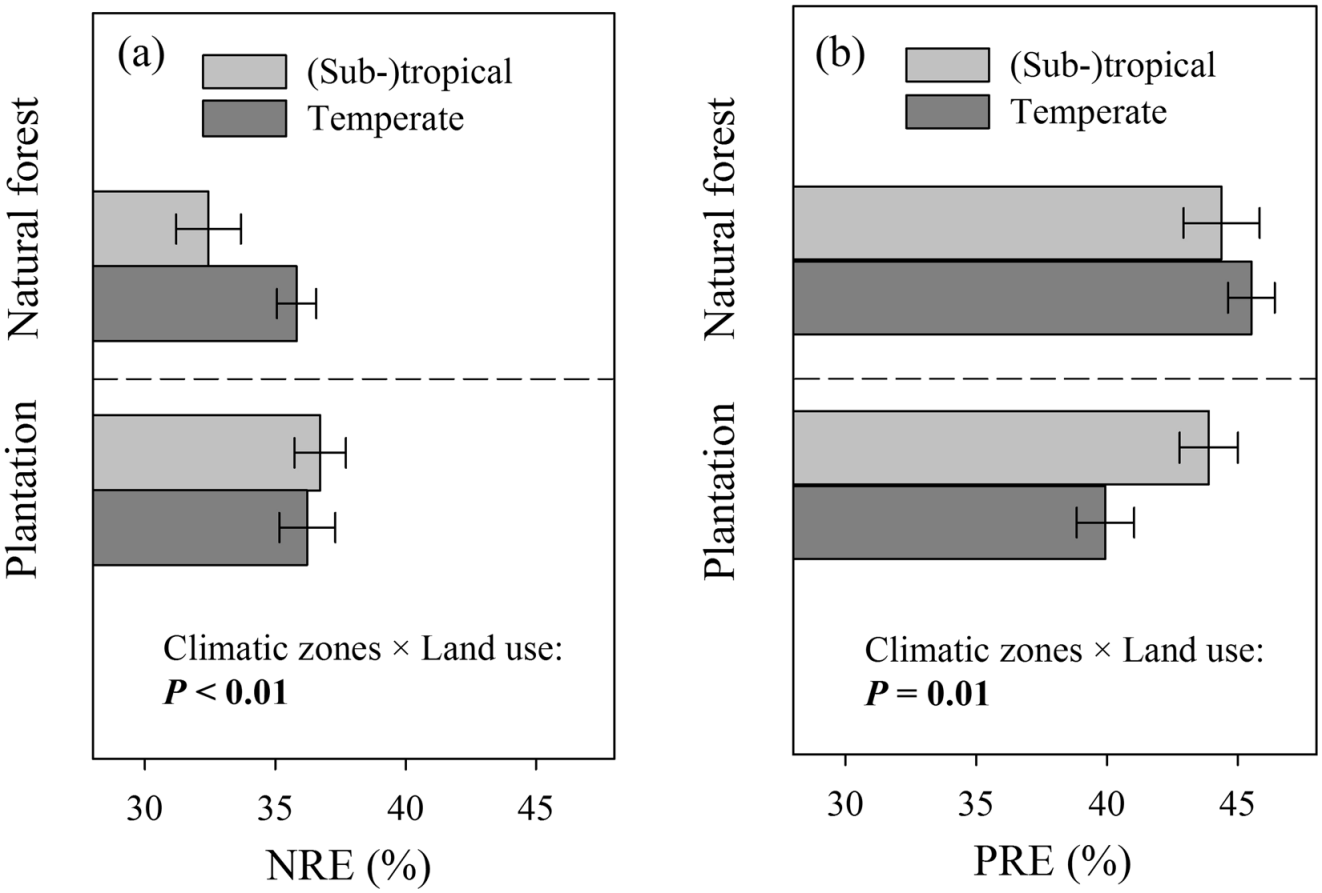
The interactions of climatic zone × land use on nutrient resorption efficiency: (**a**) NRE, and (**b**) PRE. Abbreviations are the same as Fig. 2. The error bars represent the standard error.

The interactions between climatic zones and land use were significant in explaining both NRE and PRE (Fig. 3 and Table S1). For natural forests, NRE was lower in (sub-)tropical forest than in temperate forest (Fig. 3a). In plantations, PRE was higher in (sub-)tropical regions than in temperate regions (Fig. 3b). The joint effect between soil type and forest type, as well as between land use and stand stage were significant for NRE (Table S1). The joint effects between climatic zone and stand stage, forest type and stand stage, and land use and stand stage were all significantly related to PRE (Table S1).

### Changes in NRE and PRE along climatic gradients

The results from stepwise multiple regression suggested that NRE was negatively correlated with MAP (R^2^ = 0.005, *P* = 0.057, Table 1), whereas PRE positively correlated with MAP, and negatively correlated with MAT and latitude (R^2^ = 0.007, *P* = 0.022, Table 1).

**Table 1:** Stepwise multiple regressions between NRE and PRE and mean annual temperature (MAT, °C), mean annual precipitation (MAP, mm), and latitude (LAT).

Variable	Forest type	Equation	R^2^	*P*
NRE	All	y = −0.002MAP + 38.151	0.005	**0.057**
	EBF	y = 1.851MAT−0.011MAP + 16.634	0.113	**<0.01**
	DBF	y = −0.825MAT − 0.010MAP + 53.388	0.086	**<0.01**
	ENF	y = 0.304MAT + 28.243	0.012	0.083
	DNF	y = −0.056MAP + 90.410	0.369	**<0.01**
	MF	—	—	—
	BB	—	—	—
PRE	All	y = −0.441MAT + 0.004MAP − 0.245LAT + 52.125	0.007	**0.022**
	EBF	—	—	—
	DBF	y = 0.007MAP + 38.744	0.009	**0.059**
	ENF	y = −0.553MAT + 0.012MAP + 37.999	0.024	**<0.01**
	DNF	y = −1.283MAT + 51.529	0.08	**0.056**
	MF	y = −0.281LAT + 47.908	0.019	**0.063**
	BB	y = −14.150MAT − 9.215LAT + 532.443	0.576	**<0.01**

NRE: nitrogen resorption efficiency, PRE: phosphorus resorption efficiency, EBF: evergreen broadleaf forest, DBF: deciduous broadleaf forest, ENF: evergreen needle-leaf forest, DNF: deciduous needle-leaf forest, MF: broadleaf and needle-leaf mixed forest, BB: bamboo forest. Linear models were significant when *P* < 0.05.

Within each forest type, MAT was positively correlated with NRE in EBF and ENF, but negatively correlated with NRE in DBF (Table 1). Mean annual precipitation decreased with NRE in EBF, DBF and DNF (Table 1). The resorption efficiency of P decreased with MAT in ENF, DNF and BB, and it increased with MAP in DBF and ENF (Table 1). The resorption efficiency of P was negatively related to latitude in MF and BB (Table 1).

### Changes in NRE and PRE along nutrient status

**Figure 4:**
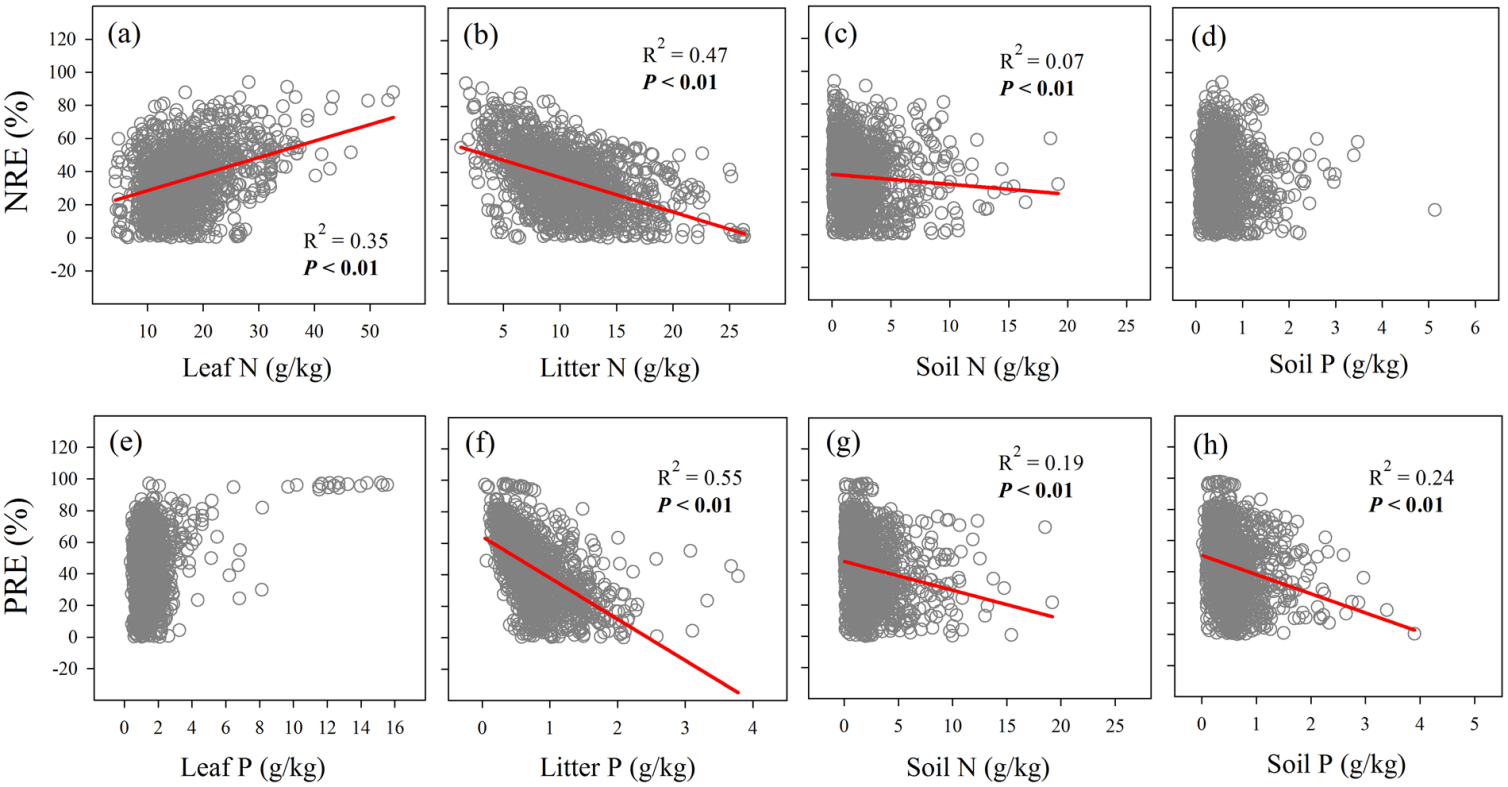
Correlations of NRE and PRE with leaf and soil nutrient status: linear relationships between (**a**) NRE vs. leaf N concentrations, (**b**) NRE vs. litter N concentrations, (**c**) NRE vs. soil N concentrations, (**d**) NRE vs. soil P concentrations, (**e**) PRE vs. leaf P concentrations, (**f**) PRE and litter P concentrations, (**g**) PRE vs. soil N concentrations, (**h**) PRE vs. soil P concentrations. N: nitrogen; P: phosphorus. Abbreviations of PRE and NRE are the same as Fig. 2.

The resorption efficiency of N increased with N concentration in leaf (N_leaf_), but decreased with N concentration in litter (N_litter_) (Fig. 4a,b). The resorption efficiency of P was not related to P concentration in leaf (P_leaf_), but decreased with P concentration in litter (P_litter_) (Fig. 4e,f). The resorption efficiency of N decreased with N concentration in soil (N_soil_) and was not related to P concentration in soil (P_soil_) (Fig. 4c,d). The resorption efficiency of P decreased with both N_soil_ and P_soil_ (Fig. 4g,h).

**Figure 5:**
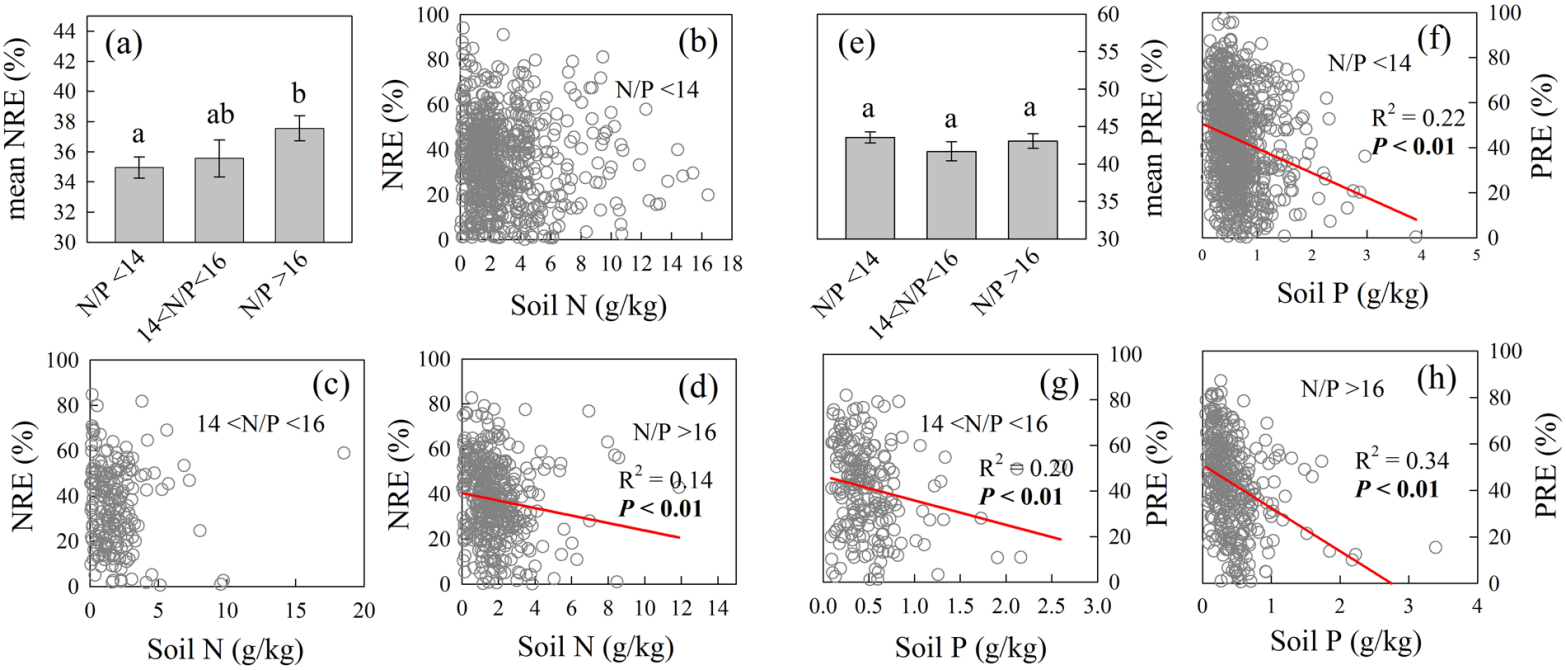
NRE and PRE across different leaf N/P ratios: (**a**) Mean NRE in leaf with three levels of leaf N/P ratio: <14, 14–16, and >16, the correlation of NRE and soil N concentrations when (**b**) leaf N/P < 14, (**c**) leaf 14 < N/P < 16, (**d**) leaf N/P > 16; (**e**) Mean PRE in leaf with three levels of leaf N/P ratio: <14, 14–16, and >16, the correlation of PRE and soil P concentrations when (**f**) leaf N/P < 14, (**g**) leaf 14 < N/P < 16, (**h**) leaf N/P > 16. Abbreviations are the same as Fig. 2.

Mean NRE was significantly higher in leaf with N/P ratio >16 than those with leaf N/P ratio <14 (Fig. 5a). No correlations were detected between NRE and N_soil_ when N/P ratio <16 (Fig. 5b,c), whereas NRE negatively related to N_soil_ when N/P ratio >16 (Fig. 5d). The resorption efficiency of P were not affected by leaf N/P ratio (Fig. 5e). The resorption efficiency of P decreased with P_soil_ across leaf N/P ratios for all vegetation types (Fig. 5f–h).

## Discussion

The resorption of N and P is an important internal-strategy for plant to retain N and P, especially under varied nutrient availability induced by global environmental changes. However, the spatial patterns and the underlying mechanisms of NRE and PRE at national scales are still under explored. In this study, we reported the patterns of NRE and PRE and their explanatory variables in the forests across China. Our results not only suggest NRE and PRE have different patterns along climatic gradients and nutrient status, but also contribute to our understanding in the nutrient strategies of China’s forests under future environmental changes, especially under increasing N deposition.

### Higher PRE than NRE across China’s forests

Our study showed the spatial patterns of NRE and PRE at the national scale and are of great significance to predict future forest productivity under global changes in China. In this study, the N and P concentrations of extant litter were used to calculate NRE and PRE in China’s forests, which were estimated to be 35.64 ± 0.48% (n = 1409) and 43.72 ± 0.55% (n = 1448), respectively (Fig. 2a,b). The results were generally lower than the mean NRE (62.1%) and the mean PRE (64.9%) at a global scale estimated by using N concentrations in senescing leaves^10^. According to the function of resorption efficiency (NRE = (1 − N_litter_/N_leaf_) × 100% and PRE = (1 − P_litter_/P_leaf_) × 100%), the reason explaining the lower resorption efficiency in our study was probably attributed to either the lower green leaf nutrient concentration or higher litter nutrient concentration. Method for sampling leaf in our study was not different from previous studies, but N and P concentrations of extant litter were likely to be higher than those of senescing leaves due to nutrient immobilization during litter decomposition^29^. In our study, the average litter N concentration was estimated to be 1.062 ± 0.011%, which was higher than that (0.974 ± 0.033%) estimated at a global scale^10^. However, the average litter P concentration (0.078 ± 0.001%) in our study was more or less than that (0.077 ± 0.006) estimated at a global scale^10^. The differences in our estimation of NRE and PRE with the calculation based on a global scale can contribute to the refinement and improvement of estimation of NRE and PRE used in models.

Some studies also reported that PRE was higher than NRE^8, 11, 30^. For example, the stoichiometric patterns of nutrient resorption in leaves at different scales were evaluated in tropical sites and a temperate forest, and the N/P resorption ratio was less than 1 (N/P resorption ratio <1)^11, 30^. The underlying mechanisms might be: (1) P is more in an inorganic form in leaf than N^14^, and therefore the process of P resorption is probably less costly; (2) P is less mobile and soluble in soil than N and is often limited in (sub-)tropical regions^3^; Higher PRE compared to NRE may imply China’s forests are more limited by P than N^30^. Mean leaf N/P ratio across China’s flora was found to be 14.4^31^ or 12.2^32^, higher than the global averages 11.0^33^ or 11.8^34^, indicating a much greater P limitation in Chinese forests than global scale; (3) N deposition can shift soil from N limitation to P limitation in temperate regions^24^, which may be another potential explanation for higher PRE than NRE in China’s forests.

### The effect of climatic zone, forest type and land use on NRE and PRE

The results from multiple regressions showed that weak correlations of NRE (R^2^ = 0.005, *P* = 0.057) and PRE (R^2^ = 0.007, *P* = 0.022) with climatic gradients, including latitude, temperature and precipitation (Table 1). Our results are inconsistent with previous studies based on the global dataset^8, 10^. If the temperate regions are limited by N and the (sub-)tropical regions are limited by P^3^, NRE should be higher in temperate regions and PRE should be higher in the (sub-)tropical regions. However, our results showed that higher NRE in temperate regions was only found in natural forests, not in plantations (Fig. 3a), and higher PRE in (sub-)tropical regions was only found in plantations, not in natural forests (Fig. 3b). Natural forests in (sub-)tropical regions generally favor N-fixing species, since these species are important N source for other species^35^. The existence of the N-fixing species reduces limitation of N in tropical forests. Our results also found that NRE and N_soil_ in (sub-)tropical natural forests were not correlated, but these two variables were negatively related in temperate forests (Fig. S4), which may be another evidence for greater N limitation in (sub-)tropical regions than in the temperate. However, plantations, no matter in (sub-)tropical or temperate regions, are often monoculture and conserves less understory layer than natural forests^36^. This probably leads to limitation of N in plantations, which could be partially reflected by the lower N_soil_ in plantations than in natural forests (Fig. S1a). Accordingly, we predict under N deposition or fertilization, a steeper decline in NRE in natural forests will probably happen in temperate regions than in (sub-)tropical regions, and a steeper decline in NRE in plantations than in natural forests. Our study also found NRE in mixed-species plantations was significantly lower than in monoculture plantations, which was probably due to a higher supply of N in mixed-species plantations, as explained by: (1) mixed-species plantations had higher mineralization rates; (2) mixed-species plantations had a smaller fraction of nutrient loss; and (3) there exists N_2_ fixation in mixed-species plantations^37^.

Our results indicated older-soil sites or mature stands conserve higher P resorption efficiency than young stands (Fig. 2b and 3b). The underlying mechanisms probably attributed to the progressive P loss or limitation along long-term development of soil or the succession of ecosystems^3^, and therefore P resorption should be a major way in the retaining of P. The increment of N caused by anthropogenic activities has been leading to more intensive P limitation in (sub-)tropical regions and a shift from N- to P-limitation in temperate regions^38^. Young stand uses other strategies to get P, including uptake of P by roots under N deposition^24^. Our results predict older-soil sites or mature stands may become more dependent on the way of resorption to conserve P under N deposition in comparison with young stand, and further investigations are needed.

Given that nutrient status and strategies acting as important factors affecting C storage in forests^1, 2^, estimations of NRE and PRE in each forest type are of great significance. In our study, both NRE and PRE followed the order: EBF < ENF < DBF < DNF (Fig. 2a,b), which indicated NRE and PRE in evergreens were lower than deciduous ones, NRE and PRE in broad-leaf species were lower than conifers. Two possible mechanisms could explain why NRE and PRE in evergreen species were lower than that in deciduous species. First, the proportion of N in insoluble protein is higher in evergreen leaves than in deciduous leaves^39^, making the process of N resorption is more energy-consuming. Second, litterfall in evergreen trees and deciduous trees occurs in different seasons. In addition, although NRE in ENF was significantly lower than that in DNF (Fig. S2c), no difference in N_soil_ was found between DNF and ENF (Fig. S2d). Lower NRE in ENF probably correlated with N_leaf_, which was found to be remarkably low (Fig. S2a). Leaf N concentration was also found to be lower in evergreens and conifers than deciduous and broadleaved species in other studies^31^. However, no significant differences were found between PRE in ENF and DNF (Fig. S3c). Leaf P concentrations in evergreen broadleaved forest and conifers were significantly lower than that in deciduous broadleaved forest^40^, and are also different in the chemical forms^41^. In addition, most conifer tree species, regardless of evergreen or deciduous, are ectomycorrhizal (ECM) trees, in which phosphatase enzyme is active in mining of organic-bound P^42^. Our results suggested NRE was more affected by plant functional type than PRE.

### The effect of nutrient status on NRE and PRE

Nutrient status, including leaf and soil nutrient status, is an important factor affecting nutrient resorption efficiency^27^. The correlation between nutrient resorption efficiency and leaf nutrient status was inconsistent in previous studies, with negative correlation^10, 26^, positive correlation^43^ and no correlations^44^. Our study found that NRE was positively correlated with leaf N concentrations at the national scale (Fig. 4a), whereas PRE did not correlate with leaf P concentrations (Fig. 4e). It is possible that the nutrient concentrations were not the determinations of nutrient resorption efficiency, but the forms of nutrient in leaf are the determinant. For example, the maximum NRE was found to decrease with increased fraction of cell walls in the leaves in a deciduous temperate forest, and the authors attributed the possible reason to a greater N occlusion in the matrix of cell wall^13^. Although higher concentrations of leaf nutrient lead to higher litter nutrient concentrations, nutrient resorption can decrease nutrient concentrations in litter^26^. The significantly negative correlations between nutrient (N and P) resorption efficiency and litter nutrient (N and P) concentrations (Fig. 4b,f) suggested nutrient resorption efficiency is a key factor regulating nutrient concentrations in litter, and therefore could further impact its decomposition rate^13, 45^.

The correlations between nutrient resorption efficiency and soil nutrient status were inconsistent in different studies^11, 27^, probably depending on whether the ecosystems were limited by nutrients. Our results indicated that NRE was negatively correlated with N_soil_ (Fig. 4c), and PRE was negatively correlated with P_soil_ (Fig. 4h), suggesting soil nutrient status is an important factor affecting site-specific nutrient resorption efficiency. Our results also found PRE was negatively correlated with N_soil_ (Fig. 4g), which suggested that soil N also can affect P recycling, and nutrient resorption is a mechanism to relieve stoichiometric imbalance^11^. However, our result was contradict with another finding, in which PRE increased with soil N content to 30 cm depth^11^. This can be attributed to the positive relationship between leaf P concentrations and soil N concentrations (Fig. S4e), which led to negative correlation between leaf N/P ratio and soil N concentrations (Fig. S4f). The effect of soil N on PRE has been tested in only few studies and the underlying mechanisms are still unclear^11^, which should be investigated in future studies.

The results suggested NRE decreased with increasing soil N concentrations only when plant was not limited by N. Our results also explained why the correlations between NRE and soil N status were inconsistent among studies^11, 27^, which was partly dependent on the relative limitation of N vs P. However, leaf N/P ratio had no significant effect on PRE (Fig. 5e), and PRE decreased with soil P concentrations across leaf N/P ratios (Fig. 5f–h). The results suggested that PRE conserves higher plasticity responding to variations of soil P status. A global meta-analysis suggested, on average, PRE decreased by 15% under P fertilization, whereas NRE decreased 12% under N fertilization^27^, which also supported that PRE had higher plasticity.

### Implications

Evaluating NRE and PRE at a large scale is of great significance based on the following aspects: (1) Investigation on nutrient resorption is a way of evaluation on nutrient limitation in forests; (2) The response of nutrient resorption can be used to evaluate the strategies of plant community to global environmental changes, especially N deposition; (3) Our results will also be useful for estimating the parameters and improving the accuracy of ecosystem and biogeochemical models. The complex and diverse forests in China provided us a perfect natural gradients of nutrient availability to evaluate the patterns and mechanisms of NRE and PRE. Overall, the different patterns of N and P resorption across China’s forests may bring about long-term effects on nutrient cycling, and thus productivity and species composition at the regional scale. The estimates of NRE and PRE in our studies should be useful for parameterization and improvement in ecosystem and biogeochemical models.

## Materials and Methods

### Study area, sampling sites and leaf, litter and soil sampling

The 1624 forest sampling sites came from a nation-wide field campaign from 2011 to 2015 (Fig. 1), which was described in detail in Tang *et al*. (under review)^46^. The size of each plot was 1000 m^2^, and each plot contained 10 subplots (10 × 10 m^2^). All the tree species in canopy layer were censused. More than five samples of mature leaves of the dominate species were collected in the growing season for analyzing N and P concentration once during 2011 to 2015. We defined the litter accumulated in the surface of the soils as “extant litter”. The extant litter and soil samples from 0–10 cm depth were collected from each plot (Fig. 1). More than five extant litter and soil samples were collected evenly along the diagonal lines of each plot. Samplings and lab analysis were conducted following a standard protocol^47^. The leaf and litter samples were dried in oven for 72 hours at 55 °C and ground in the laboratory. Soil samples were dried in air, stones, roots and other coarse residues were removed and then sieved through a 100-mesh sieve for the analyses of N and P concentrations.

### Climate variables

We obtained the long-term meteorological data, mean annual temperature (MAT), and mean annual precipitation (MAP) from the National Ecosystem Research Network of China (www.cnern.org.cn). Using the geographical coordinates of each study site, we extracted site-specific meteorological data from the nearest meteorological stations with a resolution of 30 seconds × 30 seconds (c.a. 1 × 1 km at the equator) and corrected for elevation to represent the climate at the actual sampling site.

### Measurements of the N and P concentrations

The concentrations of N and P in leaf, litter and soil (N_leaf_, N_litter_, N_soil_, P_leaf_, P_litter_ and P_soil_) were measured in the Institute of Botany (Beijing) and South China Botanical Garden (Guangzhou), Chinese Academy of Sciences. Total N concentrations in leaf, litter and soil were measured using an elemental analyzer (2400 II CHNS; Perkin-Elmer, Boston, MA, USA) at 950 °C for combustion, then reduced to 640 °C. Total P concentrations in leaf, litter and soil were measured using the molybdate/ascorbic acid method after H_2_SO_4_-H_2_O_2_ digestion^48^.

### Calculations of NRE and PRE

As the litter collected from each plot is the mixture of different species, we used the average concentrations of N and P to represent the concentrations of N and P for the plot. We used the mean N and P concentrations of the dominant tree species as leaf N and P for the plot. Therefore, plot-level NRE was calculated as:

1 $${\rm{NRE}}=(1-{{\rm{N}}}_{{\rm{litter}}}/{{\rm{N}}}_{{\rm{leaf}}})\times 100 \% $$

in which N_litter_ is the concentration of N in extant litter and N_leaf_ is the average concentrations of N in leaf of the dominant tree species at a specific plot^10^. Plot-level PRE was calculated as:

2 $${\rm{PRE}}=(1-{{\rm{P}}}_{{\rm{litter}}}/{{\rm{P}}}_{{\rm{leaf}}})\times 100 \% $$

in which P_litter_ is the concentration of P in extant litter and P_leaf_ is the average concentrations of P in the leaves of the dominant tree species for a specific plot^10^. The resorption efficiencies based on nutrient concentration were found to be not different from that based on nutrient mass^13^.

### Statistical analysis

We grouped NRE and PRE by climatic zone, soil type, forest type, land use, species richness, stand stage and leaf N/P ratio. Climatic zone was classified into (sub-)tropical (south of 30 °N) and temperate zones (north of 30 °N and Tibetan Plateau)^31^. Soil was divided into sandy, loam and clayey. The plots are also classified in different forest types, including: evergreen broadleaf forest (EBF), deciduous broadleaf forest (DBF), evergreen needle-leaf forest (ENF), deciduous needle-leaf forest (DNF), broadleaf and needle-leaf mixed forest (MF) and bamboo forest (BB) (Fig. 1). Both natural forest and plantation were included in our study. Plantation was divided into monoculture and mixed-species culture. Stand stage was divided into young, mid-age and mature stage.

Multiple Analysis of Variance Model (ANOVA) was employed to examine the effects of climatic zone, soil type, forest type, land use, species richness, stand stage and leaf N/P ratio on NRE and PRE. Multiple comparisons were conducted to compare the differences of variables among forest types. Nutrient status in this study included N and P concentrations in leaf and soil. Stepwise multiple regressions were conducted to explore the spatial patterns of NRE and PRE along climatic gradients, including latitude, MAT and MAP. Linear regressions were used to explore the correlations between NRE or PRE with N_leaf_ or P_leaf_, N_litter_ or P_litter_, N_soil_ and P_soil_. All analyses were performed using R version 3.3.3 (R Core Team (2017) (http://www.R-project.org/).

### Data accessibility

The dataset for the paper is from a national-scale field investigation campaign and will be accessible after acceptance.

## Electronic supplementary material

Supporting information
