# Supplementary material for: Different spatial patterns of nitrogen and phosphorus resorption efficiencies in China’s forests
Source: Sci Rep. 2017 Sep 6;7:10584. doi: 10.1038/s41598-017-11163-7 (PMC5587577; doi:10.1038/s41598-017-11163-7)
Supplement: Supplementary file 1 — Supporting information [file 41598_2017_11163_MOESM1_ESM.docx]

**Table S1** Multiple ANOVA results for NRE and PRE. NRE: nitrogen resorption efficiency, PRE: phosphorus resorption efficiency. ANOVA results were significant when *P* < 0.05.

|  | NRE | PRE |
| --- | --- | --- |
| Climatic zone | **0.05** | 0.84 |
| Soil type | 0.15 | 0.69 |
| Forest type | **<0.01** | **<0.01** |
| Land use | 0.17 | **<0.01** |
| Stand stage | 0.36 | **0.06** |
| Climatic zone × Soil type | 0.77 | 0.76 |
| Climatic zone × Forest type | 0.51 | **0.07** |
| Soil type × Forest type | **<0.01** | 0.71 |
| Climatic zone × Land use | **<0.01** | **0.01** |
| Soil type × Land use | 0.45 | 0.36 |
| Forest type × Land use | 0.50 | 0.31 |
| Climatic zone × Stand stage | 0.74 | **<0.01** |
| Soil type × Stand stage | 0.28 | 0.51 |
| Forest type × Stand stage | 0.59 | **<0.01** |
| Land use × Stand stage | **0.02** | **0.02** |

**Figure S1** Mean soil N and P concentrations: (a) mean soil N concentrations (g/kg) and (b) mean soil P concentrations of natural forests and plantations in (sub-)tropical and temperate regions. N: nitrogen; P: phosphorus. The effect was significant when *P* < 0.01.


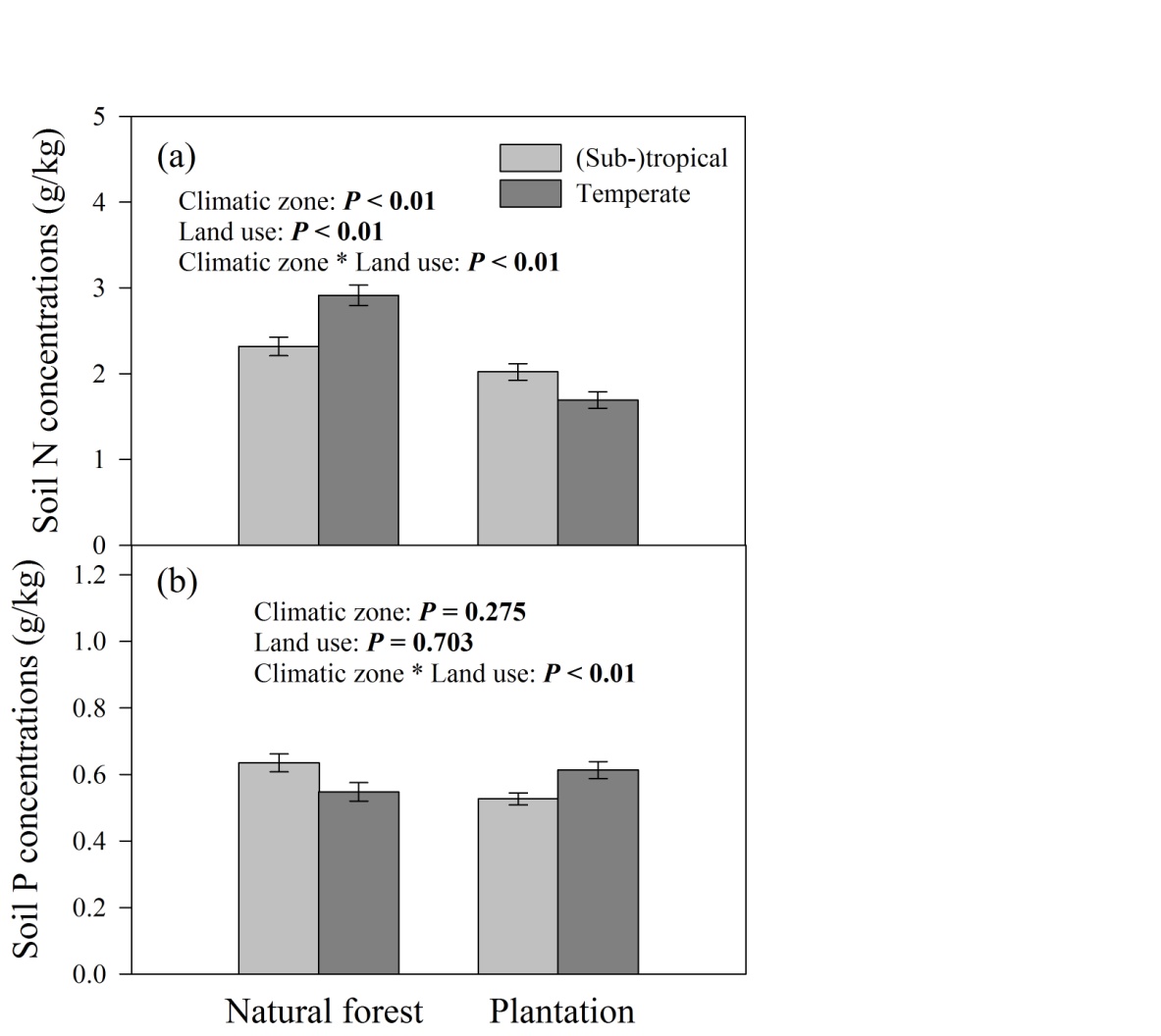


**Figure S2** N in leaf, litter and soil, and NRE: (a) Average leaf N concentrations (g/kg), (b) average litter N concentrations (g/kg), (c) average NRE (%), and (d) average soil N concentrations (g/kg) in six forest types. Abbreviations for forest types are the same as figure 1. NRE: nitrogen resorption efficiency, N: nitrogen. Different lowercase letters represent significant differences between groups.

**
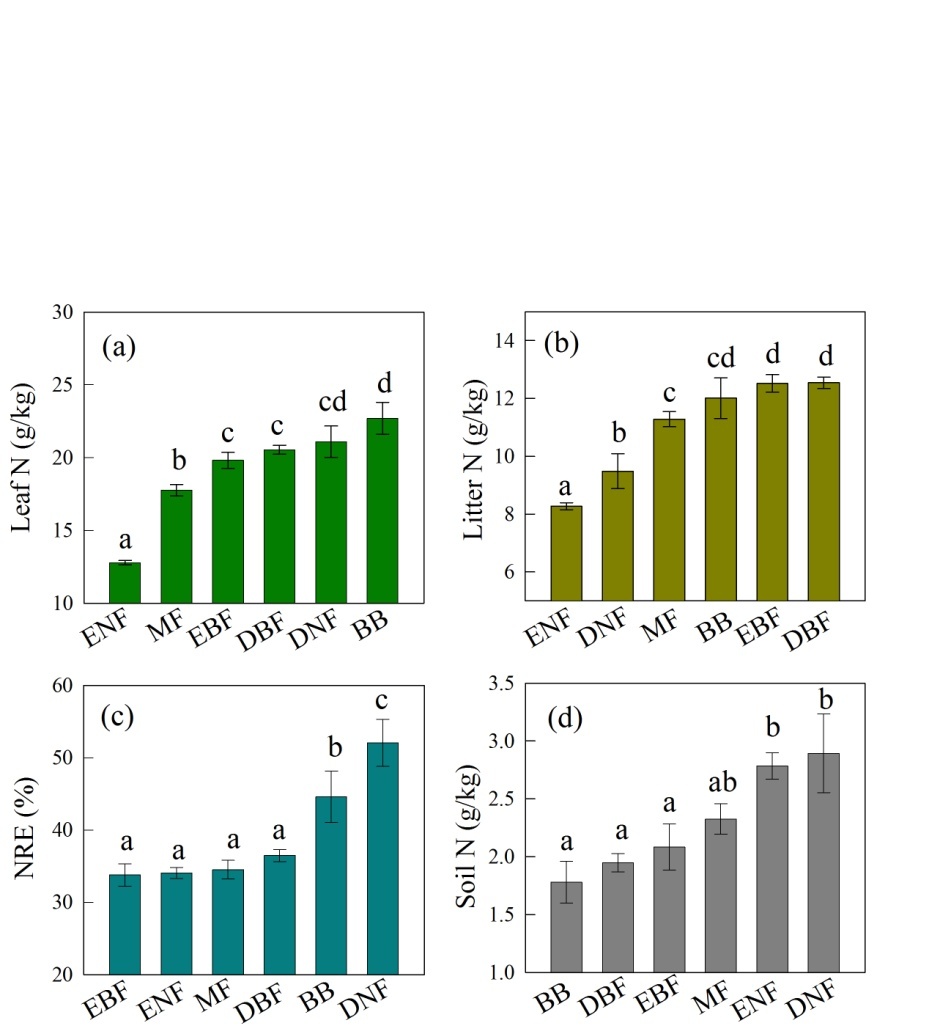
**

**Figure S3** P in leaf, litter and soil, and PRE: (a) Average leaf P concentrations (g/kg), (b) average litter P concentrations (g/kg), (c) average PRE (%), and (d) average soil P concentrations (g/kg) in six forest types. Abbreviations for forest types are the same as figure 1. PRE: phosphorus resorption efficiency, P: phosphorus. Different lowercase letters represent significant differences between groups.

**
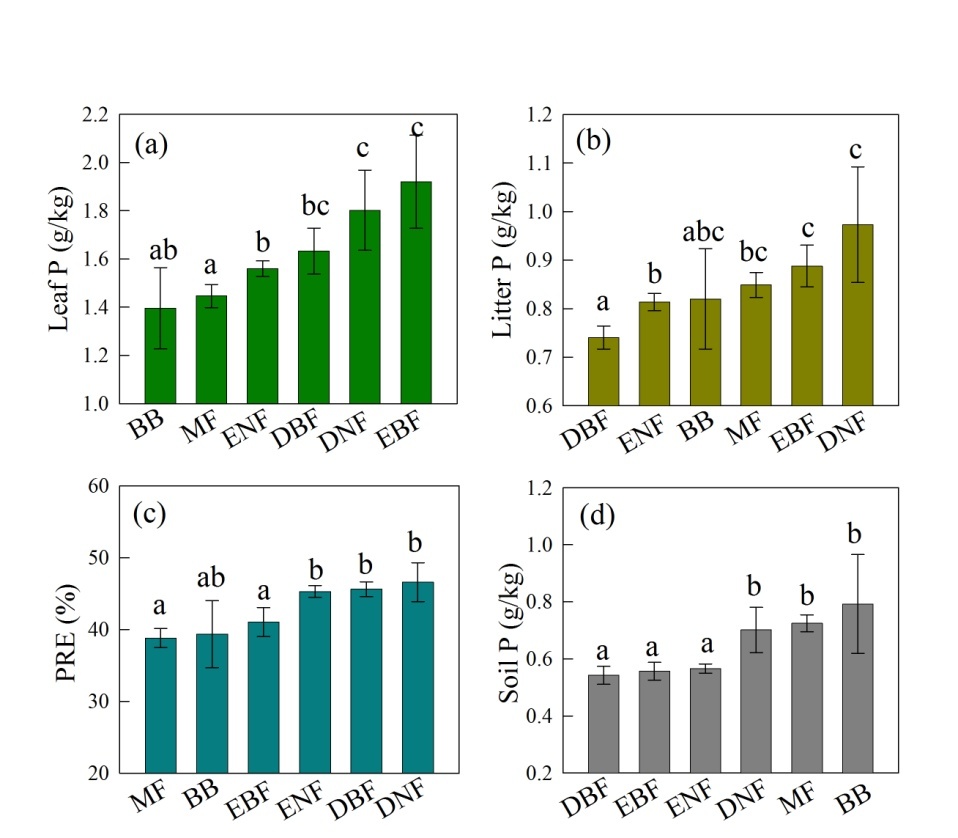
**

**Figure S4** Relationships between N and P in leaf and soil: the logarithm of soil P concentrations and (a) the logarithm of leaf N concentrations, (b) the logarithm of leaf P concentrations, (c) leaf N/P ratio; and the logarithm of soil N concentrations and d) the logarithm of leaf N concentrations, e) the logarithm of leaf P concentrations, f) leaf N/P ratio. N: nitrogen; P: phosphorus.

**
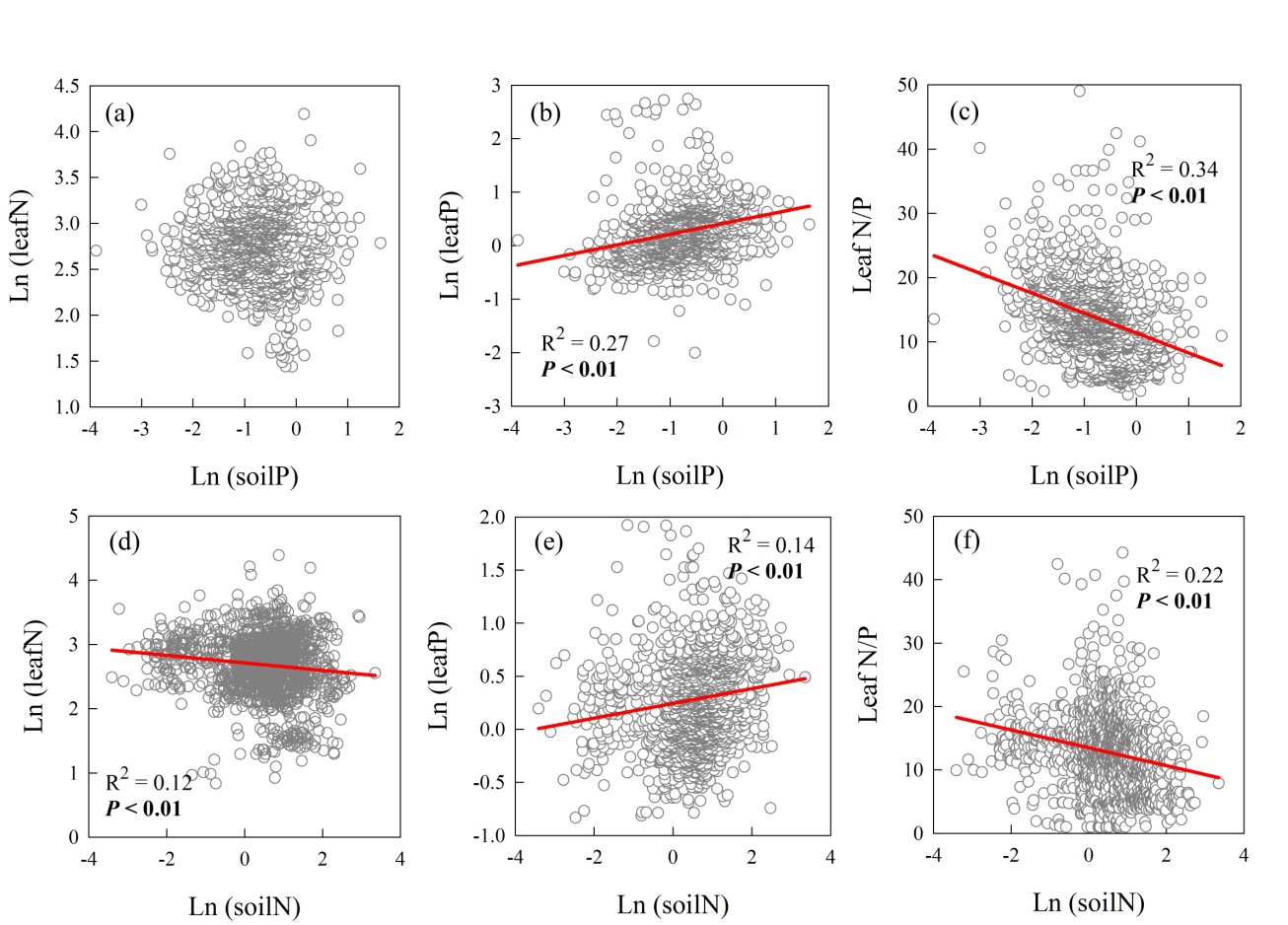
**

**Figure S5** Linear relationships between NRE vs. leaf N concentrations, NRE vs. litter N concentrations, NRE vs. soil N concentrations, NRE vs. soil P concentrations, PRE vs. leaf P concentrations, PRE and litter P concentrations, PRE vs. soil N concentrations, PRE vs. soil P concentrations. N: nitrogen; P: phosphorus. NRE: N resorption efficiency, PRE: P resorption efficiency, EBF: evergreen broadleaf forest, DBF: deciduous broadleaf forest, ENF: evergreen needle-leaf forest, DNF: deciduous needle-leaf forest, MF: broadleaf and needle-leaf mixed forest, BB: bamboo forest.

**
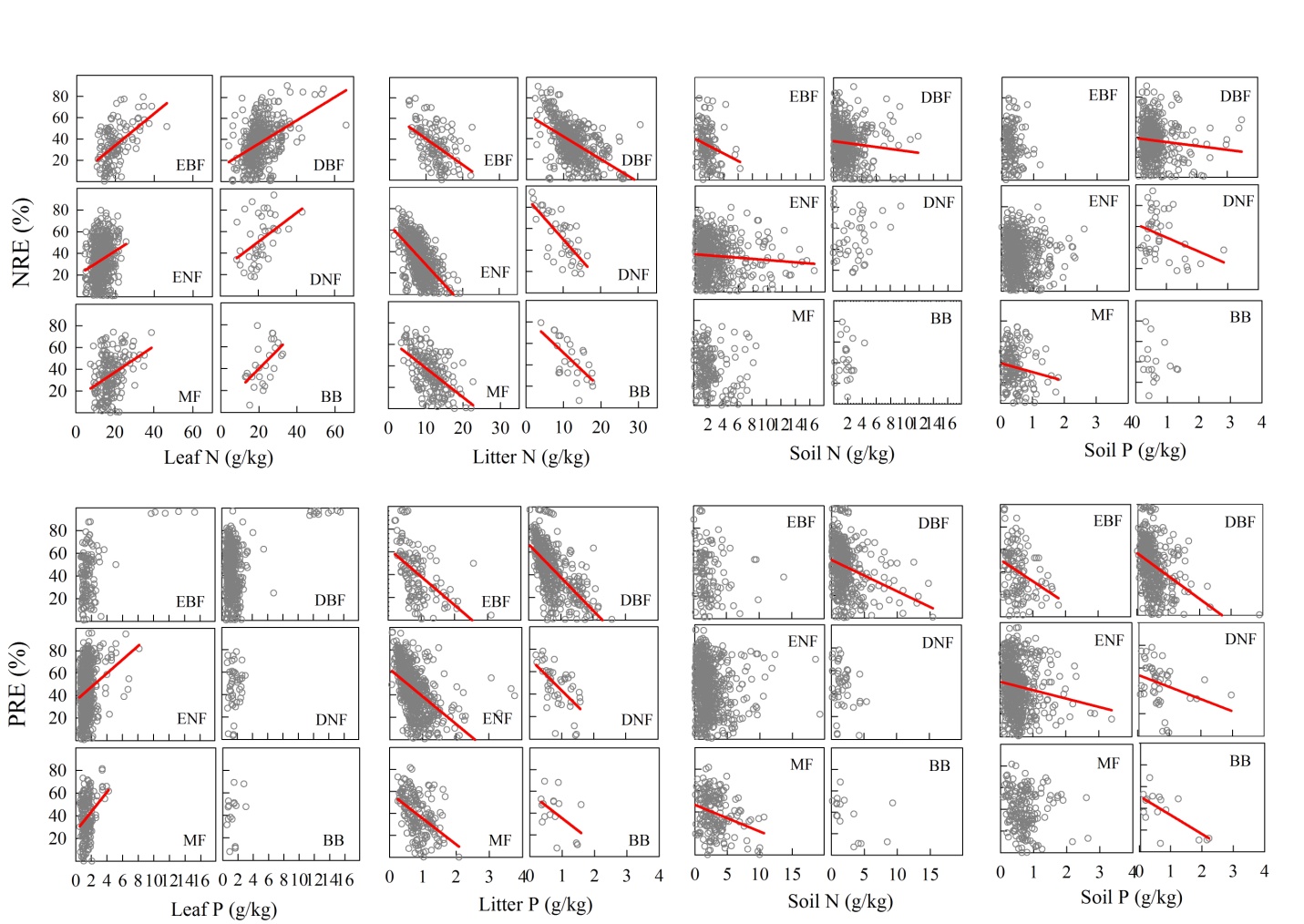
**
